# A Mixed Method to Evaluate Burden of Malaria Due to Flooding and Waterlogging in Mengcheng County, China: A Case Study

**DOI:** 10.1371/journal.pone.0097520

**Published:** 2014-05-15

**Authors:** Guoyong Ding, Lu Gao, Xuewen Li, Maigeng Zhou, Qiyong Liu, Hongyan Ren, Baofa Jiang

**Affiliations:** 1 Department of Epidemiology and Health Statistics, School of Public Health, Shandong University, Jinan City, Shandong Province, P.R.China; 2 Department of Occupational and Environmental Health, School of Public Health, Taishan Medical College, Taian City, Shandong Province, P.R.China; 3 Shandong University Climate Change and Health Center, Jinan City, Shandong Province, P.R.China; 4 Department of Environment and Health, School of Public Health, Shandong University, Jinan City, Shandong Province, P.R.China; 5 National Center for Chronic and Noncommunicable Disease Control and Prevention, China CDC, Beijing City, P.R.China; 6 State Key Laboratory for Infectious Diseases Prevention and Control, National Institute for Communicable Disease Control and Prevention, China CDC, Beijing City, P.R.China; 7 State Key Laboratory of Resources and Environmental Information System, Institute of Geographic Sciences and Natural Resources Research, Chinese Academy of Sciences, Beijing City, P.R.China; Institut Pasteur, France

## Abstract

**Background:**

Malaria is a highly climate-sensitive vector-borne infectious disease that still represents a significant public health problem in Huaihe River Basin. However, little comprehensive information about the burden of malaria caused by flooding and waterlogging is available from this region. This study aims to quantitatively assess the impact of flooding and waterlogging on the burden of malaria in a county of Anhui Province, China.

**Methods:**

A mixed method evaluation was conducted. A case-crossover study was firstly performed to evaluate the relationship between daily number of cases of malaria and flooding and waterlogging from May to October 2007 in Mengcheng County, China. Stratified Cox models were used to examine the lagged time and hazard ratios (HRs) of the risk of flooding and waterlogging on malaria. Years lived with disability (YLDs) of malaria attributable to flooding and waterlogging were then estimated based on the WHO framework of calculating potential impact fraction in the Global Burden of Disease study.

**Results:**

A total of 3683 malaria were notified during the study period. The strongest effect was shown with a 25-day lag for flooding and a 7-day lag for waterlogging. Multivariable analysis showed that an increased risk of malaria was significantly associated with flooding alone [adjusted hazard ratio (AHR)  = 1.467, 95% CI = 1.257, 1.713], waterlogging alone (AHR = 1.879, 95% CI = 1.696, 2.121), and flooding and waterlogging together (AHR = 2.926, 95% CI = 2.576, 3.325). YLDs per 1000 of malaria attributable to flooding alone, waterlogging alone and flooding and waterlogging together were 0.009 per day, 0.019 per day and 0.022 per day, respectively.

**Conclusion:**

Flooding and waterlogging can lead to higher burden of malaria in the study area. Public health action should be taken to avoid and control a potential risk of malaria epidemics after these two weather disasters.

## Introduction

Climate change is a current global concern and has an influence on the epidemiology of vector-borne diseases [1], [2]. In particular, climatic disasters play a major role in affecting the emergence and prevalence of vector-borne diseases [3]. Heavy rainfall may cause flooding or waterlogging. Flooding is an overflow of surface runoff that submerges towns and farmland, which is often caused by long-lasting heavy storms. Waterlogging is one of the most hazardous natural disasters, which can also be called as submergence, wet damage, moisture damage, and is often caused by long lasting rainfall without a heavy precipitation intensity [4]. On average, floods and other hydrological events accounted for over 50% of the disasters between 2001–2010 in the world [5]. In late June and July 2007, the persistent and heavy rainfall caused several floods in the Huaihe River Basin, China. The floods in 2007 forced an evacuation of thousands of people from homelands, with at least 89 counties and over 15.1 million people affected in Anhui Province [6]. It is important to study the impact of floods on human health for forecasting and informing the population, in order to help minimize negative consequences.

**Figure 1:**
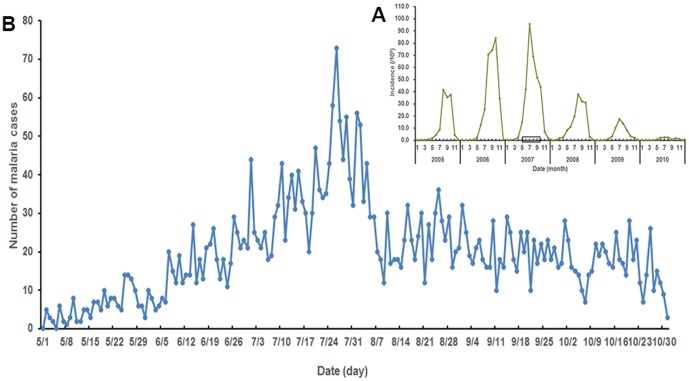
Monthly incidence of malaria from 2005–2010 (A) and daily cases of malaria during the study period (B) in Mengcheng County.

The health effects of flooding or waterlogging are complex and far-reaching, which may include increased mortality and morbidity from Malaria. Malaria, a highly climate-sensitive vector-borne infectious disease, is a major public health problem in most developing countries. At the global level, malaria is considered the world's most important vector-borne disease. According to the World Malaria Report 2012, there were an estimated 219 million cases of malaria and 660,000 deaths in 2010 [7]. Historically, a higher incidence of malaria was observed in the Huang-Huai River region of central China and the total number of malaria cases accounted for 91.2% of the total reported cases in the country in the 1970s [8]. At present, this disease still represents a significant public health problem in this region, with dramatic re-emergence since 2001 [8]. A total 27,307 malaria cases in Anhui Province were notified in the annual case reporting system with accounting for 58.5% of the total number of reported cases in China in 2007 [9]. The incidence of malaria in the northern areas of Anhui Province was higher than that in the middle and southern Anhui Province since 2000 [10]. Mengcheng, one of the northern counties of Anhui, has one of the highest burden of malaria with a peak 3,803 malaria cases reported in 2007, particularly in July and August (Figure 1) [9]. While, the rainfall in Mengcheng County brought about a severe flooding and a waterlogging before the peak incidence of cases. And it was the largest floods since the 1954 Huaihe River floods in this region [6].

Few studies have been conducted about the impact of flooding on malaria. Flooding may wash away existing mosquito-breeding sites, standing water caused by heavy rainfall or overflow of rivers can create new breeding site. This situation can result in an increase in the vector population and the potential for malaria transmission. These studies have described the disease status of malaria during post-disaster periods [11]–[14], but there was no quantitative examination on the relationship between malaria and flooding. To our knowledge, relevant studies on the association between malaria and waterlogging have not been reported. The association between these two weather events and malaria is far from clear. In addition, given little research has been conducted in China, effects of the 2007 flooding and waterlogging on malaria remain unknown. In order to know the epidemiological information on the malaria situation caused by the 2007 Huaihe River floods and to provide reliable data for the control programs in the county of Mengcheng, this study was conducted to quantify the impact of flooding and waterlogging on malaria in 2007.

## Methods

### Ethical statement

Disease surveillance data used in this study were permitted by Chinese Center for Disease Control and Prevention. All data are unidentified. The study was approved by the research institutional review board of Public Health of Shandong University.

### Study site

**Figure 2:**
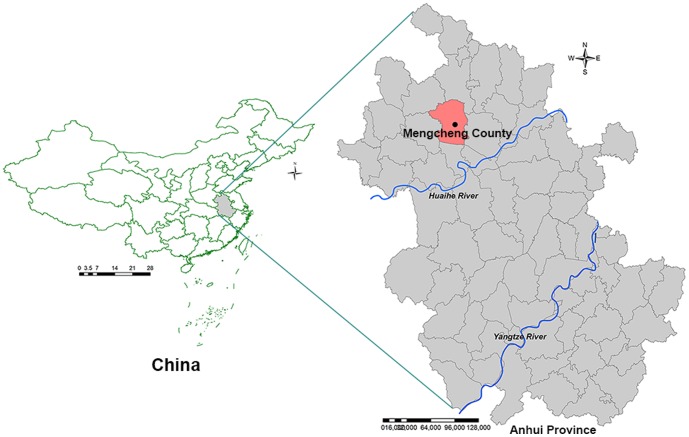
Location of the study area in Anhui Province, China.

Mengcheng County is located in Northern Anhui Province, between 32°56′ and 33°29′ of latitude north and between 116°15′ and 116°49′ of longitude east (Figure 2). Mengcheng has an area of 2091 km^2^ and a population of 1.2 million. The county is generally characterized by a sub-humid warm temperate continental monsoon with mild climate and plentiful rainfall, with an annual average temperature of 14.8°C, an annual average precipitation of 843 mm, and an annual average relative humidity of 70.4%. The geographic landscape and climate situation, such as suitable temperature and humidity, abundant rainfall, and existence of water bodies, provided favorable breeding sites for *Anopheles*. Studies conducted in the areas along the Huang and Huaihe River show that *An. sinensis* plays an important role in the *P. vivax* malaria transmission [15]–[17]. The main crops of the county are wheat, soybean, corn, and a small amount of rice. During summers, most of local residents tend to sleep outdoors.

### Data collection

#### Disease surveillance data

Malaria data were collected for the period of 2005–2010 from the National Notifiable Disease Surveillance System (NDSS). All malaria cases were defined based on the diagnostic criteria and principles of management for malaria (GB 15989-1995) issued by Ministry of Health of the People's Republic of China. Only the cases confirmed clinically and by laboratory test, including thick and thin blood smear, were included in our study. Information of cases included age, gender, type of disease, date of onset, and date of death. In China, malaria is a statutory notifiable category B infectious disease. Therefore, physicians in hospitals must report every case of malaria to the local health authority within 24 hours. Therefore, it is believed that the degree of compliance in disease notification over the study period was consistent. Demographic data were obtained from the Center for Public Health Science Data in China (http://www.phsciencedata.cn/).

#### Data on flooding and waterlogging

Meteorological events data were collected from the Yearbook of Meteorological Disasters in China and Chinese Agro-meteorological Disasters Information Data (http://cdc.cma.gov.cn/choiceStation.do). Exceptionally heavy rains occurred during the main flood season in 2007 caused serious disasters in Huaihe River Basin [6]. Mengcheng County was one of the worst affected areas. From 3 July to 9 July, the county had experienced a severe flooding which had a duration of 7 days and hit 43 thousand hectares of crops [18]. A continuous rain process during 15 July to 26 July led to a waterlogging disaster which had a duration of 12 days and hit more than 67 thousand hectares of crops [18].

#### Meteorological data

After receiving permission from National Meteorological Information Center of China, daily meteorological data were obtained from the China Meteorological Data Sharing Service System (http://cdc.cma.gov.cn/). The meteorological variables included daily average temperature, daily average relative humidity, daily rainfall, and daily sunshine duration. Because the effect of meteorological variables on the incidence of malaria is not linear [19]–[21], average temperature, average relative humidity, rainfall and sunshine duration were transformed to categorical variables. Average temperature was grouped into three levels: <20°C, 20–30°C, and >30°C. Average relative humidity was grouped into <60%, 60–80%, and >80%. Such categorization of temperature and relative humidity was based on the reports of the climatic conditions thought to be suitable for transmission malaria by *Anopheles* [21]–[25]. According to the scale of precipitation in China, rainfall was grouped into four levels in our study: light rain (0.1–9.9 mm per day), moderate rain (10–24.9 mm per day), heavy rain (25–49.9 mm per day) and rainstorm (>50 mm per day) [26]. Sunshine duration was grouped into two levels based on median of variables: <6 h and ≥6 h.

### Study design and statistical analysis

A method combining a case-crossover study and attributable burden of disease was adopted to carry out the risk assessment on malaria caused by flooding and waterlogging. Firstly, a descriptive analysis was performed to describe characteristics of cases of malaria and the distribution of meteorological factors. Secondly, a case-crossover study, which was proposed for the study of transient outcomes that were impacted by short-term events or exposures [27], [28], was conducted to examine whether flooding and waterlogging were related to the number of malaria cases. The case-crossover design, which is a special case-control design where every case serves as its own control, offers the ability to control confounders by design rather than by modeling [29], [30]. Therefore, potential confounding due to age, sex, personality, and other fixed characteristics is eliminated [31]. The period of May- October 2007 was selected as our study period. The 1:3 symmetric bidirectional design was applied for selecting references to overcome the time trend of exposure and confounding, because referents were within the same season and on the same day of the week as the index time [32]. Six referents 7, 14 and 21 days before and after the event day were selected as control days. For example, when the event day was 9 July 2007, we considered 18 June, 25 June, 2 July, 16 July, 23 July, and 30 July 2007 as reference days.

The association between the number of cases of malaria and flooding and waterlogging was estimated by using the hazard ratios (HRs) and their 95% confidence intervals (CI) on the basis of stratified Cox models. We used fitting a stratified Cox model with the “Breslow” option for handing tied failure times [33]. The effects of exposure to flooding and waterlogging were explored for the duration of the effect-period using univariate stratified Cox models. The lagged 0 day was labeled “L0”, and the lagged 1 day was “L1”. The lagged 2 days was “L2”, and so forth. Meteorological events may indirectly lead to an increase in vector-borne diseases through the expansion in the number and range of vector habitats [34]. Under suitable conditions, the duration from egg development to adult mosquitoes is about 9-15 days. The incubation period of *P. vivax* malaria ranges from 6–21 days. Thus, the lag effect up to 60 days was assessed by the stratified Cox regression analysis. The maximum lag time was selected based on the maximum HRs (at this point, the best estimate of duration had minimal nondifferential misclassification [27]). After adjusting for average temperature, average relative humidity, rainfall, and sunshine duration in multivariate stratified Cox models, HRs and 95% CI of malaria due to the exposure to flooding and waterlogging were calculated in each model. All statistical analyses were performed using SAS 9.1.3 (SAS Institute Inc., USA).

Thirdly, years lived with disability (YLDs) were calculated to estimate the burden of disease due to malaria during exposure effect-period of flooding and waterlogging. Since there was no death of malaria notified during the study period, we adopted YLDs to estimate the burden of disease with the consideration of lagged effects. The method of estimating YLDs as recommended by the World Health Organization (WHO) was used to calculate burden of malaria during exposure effect-period of flooding and waterlogging [35]. Calculations of YLDs and YLD per 1000 were made using DisMod II (WHO, 2001) and Microsoft Office Excel 2003 (Microsoft Corp., USA).

Lastly, the potential impact fraction (PIF) and attributable YLDs were estimated for the percentage of burden of disease due to malaria that was attributed to flooding and waterlogging. The following formula for PIF was 
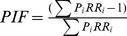
 [36].

Where: P_i_ = Proportion of the population in exposure category i. RR_i_ = Relative risk at exposure category i compared to the reference level (If the rare disease assumption holds, HR is a good approximation to RR [37]).

The YLDs for the population were multiplied by PIF to calculate the fraction of malaria attributable to flooding and waterlogging for the study population, as shown in the following equation. 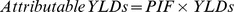 [36].

## Results

### Descriptive analysis for the disease and meteorological data

**Figure 3:**
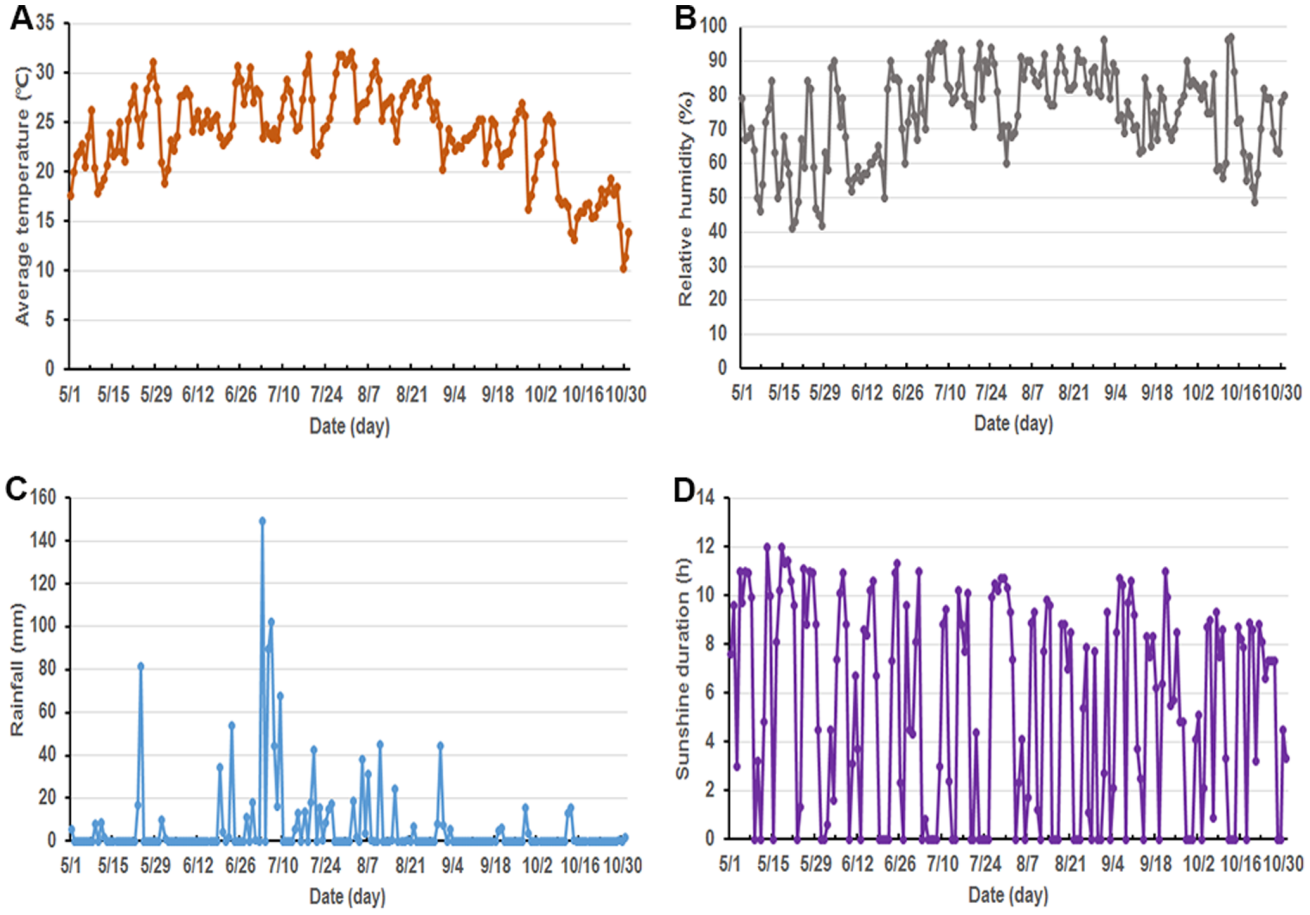
The distribution of daily meteorological factors during the study period. (A) Daily average temperature in Mengcheng County; (B) daily average relative humidity in Mengcheng County; (C) daily rainfall in Mengcheng County; (D) daily sunshine duration in Mengcheng County.

From 2005 to 2010, a total of 11491 malaria were reported in the study area with a mean monthly incidence rate of 13.76/10^5^. The monthly incidence peaked in July 2007, reaching 95.78/10^5^. A seasonal distribution of incidence was observed with most cases occurred in summer and autumn (Figure 1-A). There were 3683 (2146 males and 1537 females) notified malaria cases during the study period, accounted for 96.2% of the total reported cases in this county in 2007. By age groups: 22.7% aged below 14 years, 55.4% aged between 15 and 59 years, and 21.9% aged over 60 years. There was a distinct difference in the daily number of malaria cases among different months with a peak from late July to early August, and the maximum number of malaria cases reached 73 on 26 July (Figure 1-B). Figure 3 shows the distribution of daily meteorological factors during the study period. The mean daily average temperature over the study period was 23.9°C (Figure 3-A) and mean daily average relative humidity was 74.3% (Figure 3-B). The total precipitation in Mengcheng was 1177 mm from May to October with maximum rainfall occurred on 3 July (Figure 3-C). Besides, the mean daily sunshine duration was 5.3 h (Figure 3-D).

### Analysis for lagged effects

**Figure 4:**
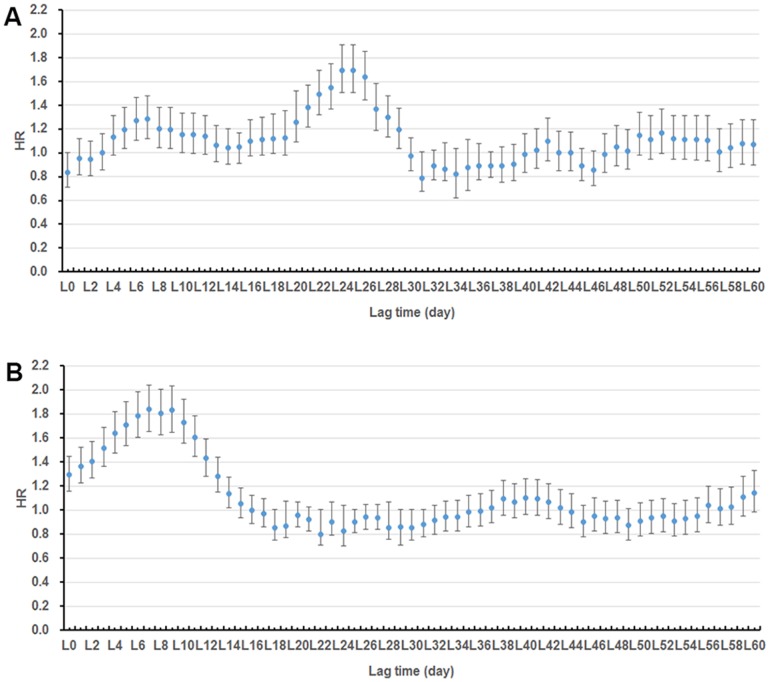
HR estimates of flooding (A) and waterlogging (B) on the risk of malaria in different lagged days.

**Figure 5:**
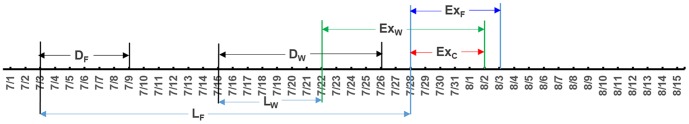
A timeline of exploring for duration of the effect-period. D_F_: duration of flooding; D_W_: duration of waterlogging; L_F_: lag time of flooding; L_W_: lag time of waterlogging; Ex_F_: the exposure effect-period for flooding alone; Exw: the exposure effect-period for waterlogging alone; Ex_C_: the common exposure effect-period for both flooding and waterlogging.

Results from the lag time analysis are summarized in Figure 4. Flooding significantly increased the number of cases from L5 to L9 and from L20 to L29 (HRs>1, *P*<0.05), and waterlogging was from L0 to L14 (HRs>1, *P*<0.05). The strongest effects were observed at L25 days (HR = 1.695, 95% CI = 1.505, 1.910) for flooding, and L7 days (HR = 1.838, 95% CI = 1.654, 2.042) for waterlogging, respectively. After adjusting the durations of exposure and lagged effects, exposure effect-period for flooding was from 28 July to 3 August, and effect-period for waterlogging was from 22 July to 2 August (Figure 5). Common exposure effect-period for both flooding and waterlogging was from 28 July to 2 August (Figure 5).

### Multivariate analysis

HRs of flooding and waterlogging on the risk of malaria are presented in Table 1. Flooding alone was significantly associated with an increased risk of malaria with adjustment for meteorological factors (AHR = 1.467, 95% CI = 1.257, 1.713). The AHR of malaria for waterlogging alone was 1.879 (95% CI = 1.696, 2.121) in the multivariate model. The risk for malaria was significantly associated with flooding and waterlogging together (AHR = 2.926, 95% CI = 2.576, 3.325).

**Table 1:** AHRs of flooding and waterlogging on the risk of malaria in multivariate stratified Cox models.

	AHR (95% CI)		
Model	Flooding alone	Waterlogging alone	Both flooding and waterlogging
Model 1^a^	1.687 (1.498–1.901)	1.837 (1.653–2.041)	2.642 (2.335–2.988)
Model 2^b^	1.695 (1.505–1.910)	1.818 (1.635–2.020)	2.905 (2.568–3.286)
Model 3^c^	1.515 (1.297–1.768)	1.919 (1.717–2.146)	2.366 (2.116–2.647)
Model 4^d^	1.687 (1.487–1.914)	1.837 (1.651–2.042)	2.395 (2.131–2.691)
Model 5^e^	1.467 (1.257–1.713)	1.897 (1.696–2.121)	2.631 (2.341–2.956)

AHR: adjusted hazard ratio; CI: confidence intervals.

a adjusted for average temperature; ^b^adjusted for average relative humidity; ^c^adjusted for rainfall; ^d^adjusted for sunshine duration; ^e^adjusted for average temperature, average relative humidity, rainfall, and sunshine duration.

### Analysis for attributable YLDs of malaria

Tables 2, 3 and 4 display the burden of disease due to malaria caused by flooding and waterlogging. As shown in Table 2, the incidence rate and YLD per 1000 of malaria caused by flooding alone between 2 August and 3 August were 4.569/10^5^ and 0.028, respectively. The YLD per 1000 of male at this stage was higher than that of female (0.032 vs. 0.024). The YLD per 1000 of malaria was highest in old people above 80 years of age (0.090), followed by the 60–69 years old age group (0.050). Table 3 shows that the incidence rate and YLD per 1000 of malaria caused by waterlogging alone between 22 July and 28 July were 25.604/10^5^ and 0.242, respectively. The YLD per 1000 of male was also higher than that of female (0.273 vs. 0.209). The age of 5–14 years had the highest YLD per 1000 (0.706), followed by the age of 60–69 years (0.496). The burden of disease due to malaria caused by flooding and waterlogging together from 28 July to 2 August is given in Table 4. The incidence rate and YLD per 1000 of malaria were 19.483/10^5^ and 0.168, respectively. The YLD per 1000 for malaria in male (0.182) was higher than that in female (0.153). The highest YLD per 1000 of malaria was in old people above 80 years of age (0.351), and the second was in people aged between 70 and 79 years (0.305).

**Table 2:** The epidemiological burden of malaria caused by flooding alone during exposure effect-period for flooding.

			YLD per 1000		
Age (years)	Case	Incidence (1/10^5^)	Males	Females	Persons
0–4	4	4.810	0.019	0.054	0.036
5–14	9	5.554	0.020	0.040	0.028
15–29	6	1.829	0.010	0.021	0.015
30–44	15	4.773	0.043	0.025	0.034
45–59	7	4.665	0.047	0.000	0.024
60–69	6	9.257	0.064	0.033	0.050
70–79	4	8.983	0.059	0.018	0.038
80+	2	15.319	0.211	0.020	0.090
Total	53	4.569	0.032	0.024	0.028

YLD: year lived with disability.

**Table 3:** The epidemiological burden of malaria caused by waterlogging alone during exposure effect-period for waterlogging.

			YLD per 1000		
Age (years)	Case	Incidence (1/10^5^)	Males	Females	Persons
0–4	16	19.240	0.246	0.137	0.194
5–14	50	30.856	0.803	0.573	0.706
15–29	44	13.412	0.151	0.138	0.145
30–44	60	19.091	0.097	0.075	0.086
45–59	51	33.989	0.168	0.175	0.171
60–69	44	67.888	0.460	0.537	0.496
70–79	24	53.896	0.389	0.281	0.333
80+	8	61.275	0.072	0.289	0.209
Total	297	25.604	0.273	0.209	0.242

YLD: year lived with disability.

**Table 4:** The epidemiological burden of malaria caused by flooding and waterlogging together during exposure common effect-period for both flooding and waterlogging.

			YLD per 1000		
Age (years)	Case	Incidence (1/10^5^)	Males	Female	Persons
0–4	9	10.822	0.071	0.042	0.057
5–14	37	22.834	0.251	0.306	0.274
15–29	34	10.364	0.122	0.068	0.097
30–44	46	14.637	0.076	0.077	0.077
45–59	45	29.991	0.241	0.214	0.228
60-69	26	40.115	0.503	0.064	0.298
70–79	20	44.914	0.207	0.396	0.305
80+	9	68.934	0.091	0.503	0.351
Total	226	19.483	0.182	0.153	0.168

YLD: year lived with disability.

**Figure 6:**
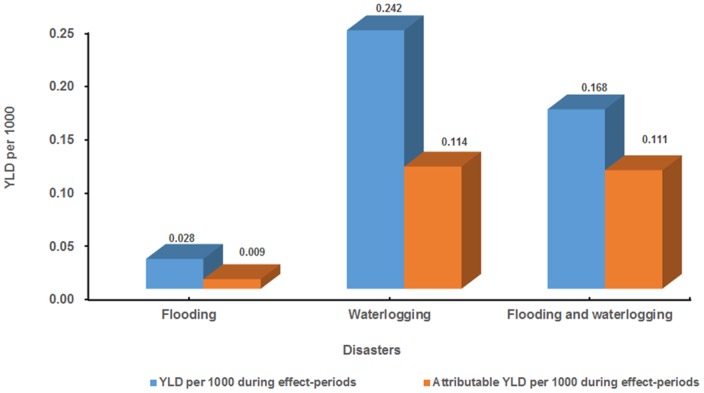
YLD per 1000 and attributable YLD per 1000 of malaria caused by flooding and waterlogging during exposure effect-period.

We assumed proportion of the study population in exposing these two disasters at 100 percent (i.e. P_i_ = 1). Based on the estimates of HRs and the formula of PIF above, PIFs of the study population exposed to flooding alone, waterlogging alone, and flooding and waterlogging together were 0.318, 0.473, and 0.658, respectively. The PIFs were considered in the further calculation of attributable YLDs. Figure 6 shows YLD per 1000 and attributable YLD per 1000 of the study population during different exposure effect-period of disasters. The attributable YLD per 1000 during common exposure effect-period for both flooding and waterlogging (0.111/5 = 0.022 per day) was higher than that exposed to flooding alone (0.009/1 = 0.009 per day) and waterlogging alone (0.114/6 = 0.019 per day).

## Discussion

Our results indicate that flooding and waterlogging play an important role in the epidemic of malaria during the flood season. This was first time that the study quantified the association between malaria and flooding or waterlogging using a mixed method in Mengcheng County of Anhui Province, China. The study confirms that exposure to flooding and waterlogging will affect burden of malaria. Although the study is based on only one area in Anhui Province, the real burden of malaria due to flooding and waterlogging will be much higher than the estimates from this study, given the larger population at risk in China. Determining the effect of these two events on burden of disease due to malaria would be beneficial for malaria risk assessment and thus providing a basis for the policy making for malaria control technologies.

Increased numbers of cases of malaria have been noted after floods in some countries. In Africa, an epidemiological study found that the incidence of endemic malaria increased four to fivefold following the 2000 flooding in Mozambique [38]. Another study reported that malaria was one main impact of flooding on human health in Gambella region [39]. After flooding, there was an increased risk of malaria epidemics in Khartoum [40]–[42]. WHO found that the flooding in the Dominican Republic in 2004 led to malaria outbreaks [34]. For other countries, flooding has also been associated with changes in habitat that were beneficial for breeding and preceded an extreme rise in malaria cases [43]–[46]. Additionally, periodic flooding linked to El Niño has been associated with malaria epidemics in Peru, Bolivia and the USA [47]–[49]. Similar findings have been reported in our study. Our study shows that the risk for malaria epidemics following flooding is very high. Malaria is sensitive to environmental change. Climatic variables have been established as important environmental drivers of malaria transmission [22], because climatic factors can impact on the growth and reproduction rates of mosquitoes, the temporal activity pattern of the population as well as the life cycle of *Plasmodium* [50], [51]. After fitting meteorological factors, flooding and waterlogging were significantly associated with an increased risk of malaria in our study, which was not just due to the seasonal fluctuation. Results indicate that flooding and waterlogging played an important role in the peak of malaria incidence from late July to early August in 2007.

Evidence of malaria associated with extreme dry weather is mixed. In South America, one study found that malaria mortality is strongly related to drought in the year before outbreaks [52]. Another study showed that droughts favor the development of malaria epidemics in Colombia and Guyana, and epidemics lag a drought by 1 year in Venezuela [53]. However, there are some studies showing decreases in risk of malaria associated with droughts. Studies from the Sahel revealed that the decreases in malaria prevalence and incidence are likely due to the disappearance of the *An. funestus* as a result of severe droughts [54], [55]. Sultan's study showed that malaria cases are rare in the dry season and during drought [56]. Another study of *Plasmodium falciparum* transmission by *An. arabiensis* and *An. funestus* during a period of drought (2004–2005) in Zambia reported reduced mosquito activity and reduced numbers of malaria cases during the period of drought [57]. Our study area has a sub-humid warm temperate continental monsoon with mild climate and plentiful rainfall. No extreme dry weather occurred during the study period.

This study has identified a longer lagged effect of flooding on malaria than that of waterlogging. Standing water caused by heavy rainfall or overflow of rivers in the flooding-period or waterlogging-period can create new breeding sites. This situation can result (with typically some weeks' delay) in an increase of the vector population and potential for disease transmission [58]. In this study, the strongest lagged effect of the flooding was observed at 25 lagged days. During the early flooding, floodwaters may wash away breeding sites and, hence, no increasing mosquito-borne transmission [59]. But mosquito breeding comes back when the waters recede. The flooding in 2007 may indirectly lead to an increase in malaria through the expansion in the number and range of *An. sinensis*. Considering the incubation periods of the parasite in the mosquito and the human, the lagged time of 7 days between waterlogging and increased malaria transmission is not biologically feasible. Thus, we assume that the waterlogging in 2007 may indirectly affect malaria through providing proper environmental conditions for adult mosquitoes' activity, because activity of adult *An. sinensis* had a certain bearing on rainfall, humidity and air temperature [60].

The hazard ratio and attributable YLDs of flooding and waterlogging together were higher than those of flooding alone and waterlogging alone, which suggests that burden of disease due to malaria caused by flooding and waterlogging together is more severe than their individual independent burden alone. The environment during exposure effect-period of the flooding and waterlogging together may indirectly lead to an increase in malaria through the expansion in the number, range and activity of vector habitats [34]. Standing water caused by heavy rainfall or overflow of rivers can act as breeding sites for *An. sinensis*, and therefore enhance the potential for exposure of the disaster-affected population and emergency workers to increasing risk of malaria [34]. Suitable temperature and rainfall during waterlogging period forced wild *An. sinensis* into indoor residential spaces and increased the chance to be bitten by mosquitoes. This indicates that flooding and waterlogging together can make large-scale ecological changes for creating an environment favorable for the more *An. sinensis* and increasing the survival and longevity of the adult *An. sinensis*.

This study has also indicated that burden of malaria caused by waterlogging alone is more severe than that by flooding alone. It is biologically plausible that moderate rainfall and moist environment during the waterlogging-day increase adult mosquitoes' activity and susceptible people become sick easily after being bitten by adult *An. sinensis* [10], [60]. While excessive floodwater during the flooding-day may partly destroy breeding sites and flush out the mosquitoes larvae [61]. This effect partially detracted burden of malaria caused by flooding. Additionally, we found that burden of disease due to malaria among males was more than females, and people who are older and children were vulnerable groups of malaria. This may be because that males participated in more relief work and engaged more frequently in emergency than females did, leading to a higher exposure to adverse environment among males [62]. In addition, there is a custom that Chinese men remove their shirts when it is hot and suffocating weather during summer in China. Children have immature immune system, and older people may have weak immune systems to in responding to malaria. Hence, males, older people and children are the population groups that are most vulnerable for malaria after flooding and waterlogging.

There are some strengths in applying a mixed approach. Firstly, based on our approach, the attributable burden of disease caused by meteorological conditions could be estimated explicitly, which could be borrowed and validated by other studies in this field. Secondly, the symmetric bidirectional case-crossover design can avoid bias resulting from time trend in the exposure series [63], and can quantitatively assess the risk of the spread of infections caused by environmental factors. Thirdly, we have controlled other meteorological factors in the multivariate models with consideration of lagged effects of flooding and waterlogging.

There are some limitations in our study. Firstly, not all environmental factors were taken into account for analysis the risk of malaria. As with other vector-borne diseases, malaria typically was driven by climatic, ecological and human factors. We have only analyzed the effect of flooding and waterlogging on malaria after adjusting climatic factors. Other factors, e.g. human activities, mosquitoes' activity, availability of health services, could not be included in this analysis. Secondly, the malaria data were from the NDSS and under-reporting bias is inevitable. Some people with mild clinical symptoms and self-treated cases might not seek medical help. This could lead to an underestimation of attributable YLD due to malaria. Thirdly, only two meteorological events and one study area in Anhui are selected in the analysis. Moreover, the transmission of malaria is very complicated, and more studies in other floods affected regions in China with different climatic, ecological and human conditions are still needed to assess the risk from ecology.

## Conclusions

A key conclusion of this study is that flooding and waterlogging contribute to unusually high incidence of malaria in the study region. In addition, risk of malaria caused by both flooding and waterlogging is greater than their individual risk alone. Therefore, effective preventive and treatment interventions should be developed to avoid and control a potential risk of malaria epidemics after flooding and waterlogging. Particular vulnerable groups, including males, older people and children, should be paid more attention in developing strategies to prevent and reduce the health impact of flooding and waterlogging.
